# Saturdays-in-Motion: Education and Empowerment through an Interdisciplinary Team Approach for Parkinson's Disease in Cali-Colombia

**DOI:** 10.1155/2020/2497386

**Published:** 2020-07-15

**Authors:** Beatriz E. Muñoz, Valentina Quintana-Peña, Maria C. Gonzalez, Jaime A. Valderrama, Yor Jaggy Castaño-Pino, Domiciano Rincón, Andrés Navarro, Jorge L. Orozco

**Affiliations:** ^1^Fundación Valle del Lili, Cali, Colombia; ^2^Grupo de Investigación I2T, Cali, Colombia; ^3^Universidad Icesi, Cali, Colombia; ^4^Centro de Investigaciones Clínicas [CIC], Fundación Valle del Lili, Cali, Colombia

## Abstract

**Introduction:**

Parkinson's disease (PD) is one of the most prevalent age-related neurodegenerative disorders. The progression of PD produces an important disease burden in patients due to functional impairment, which also has repercussions on caregivers. In addition, it has become a challenge for health systems, especially in developing countries, which have limited resources. Multidisciplinary teams with a community approach have proved effective in high-income countries; however, there is no reported literature in low- and middle-income countries about this kind of initiative.

**Objective:**

This paper aims to document the experience of patients, caregivers, and experts in a community approach as an innovative model in a middle-income country.

**Methods:**

A quantitative descriptive research was conducted. The selection criteria were having a PD diagnosis, attending with a caregiver to Saturdays in Motion (SIM), or being a clinical expert invited to SIM. PD patients and their caregivers answered three surveys on their points of view with respect to SIM: SIM and their quality of life (QoL) and PDQ-39 and Zarit, whereas clinical experts completed two questions related to the SIM program. Descriptive statistics were used to summarize the results of the surveys and clinical tests.

**Results:**

Forty-eight, twenty-four, and twenty-one subjects answered surveys one, two, and three, respectively. In total, four clinical experts were interviewed. 87.9% of the patients consider that SIM activities improved their QoL. The most affected areas in PDQ-39 were those related to the social area. Around 66.6% of the caregivers reported a mild burden on Zarit and think that SIM enhances the PD patient's QoL. Clinical experts highlighted the sense of community and empathy.

**Conclusion:**

Our preliminary experience shows a multidisciplinary model with a community approach which redefines the traditional relationship between patients, caregivers, and clinical experts. This aim of this initiative is that education and empowerment patients and caregivers reach a better perception of QoL.

## 1. Introduction

Worldwide, Parkinson's disease (PD) is the second most frequent neurodegenerative disease and its prevalence increases with aging. It has been predicted that by 2030 the population of people living with PD will be no less than twice that of the current population [1]. In Colombia, the approximate prevalence of PD is 47 cases per 100,000 inhabitants [2].

First, PD generates an economic burden, and Colombia has reported expenses of $10,7 million USD per year for patients with PD. This negatively affects the economy of countries, especially developing economies such as Colombia where per capita income is about $ 8,000 USD per year (seven times lower than the USA) [2]. In the USA, the estimated burden of the associated direct and indirect costs, five years after PD diagnosis, was reported to be approximately $ 26,096 USD higher than the matched control [3]. In Colombia, there is no information about the costs of the caregiver's burden.

Second, there is the social burden, as PD proceeds its natural course linked with motor complications such as tremor, rigidity, bradykinesia, and dyskinesia. Furthermore, nonmotor symptoms, such as depression, anxiety, dysphagia, bowel and bladder dysfunction, sleep disorders, and communication impairments, among other symptoms, interfere with everyday activities. It can also cause social image distortion, shame, and anxiety about the prognosis and the future, thereby affecting a patient's quality of life (QoL) [1, 4]. According to the Global Burden of Disease Study, in 2016, PD caused 3.2 million disability-adjusted life years [5]. Even though caregivers usually have better health, they may be exposed to high levels of emotional stress and burnout [4, 6] related to the giving up of a career, leisure activities, and/or social activities, in order to take care of the person with PD [7]. Additionally, the literature reports a lower quality of life and mental health for the caregivers than for the general population [8, 9], which may decrease the effectiveness of the care provided to PD patients [10, 11].

In recent years, innovative methodologies have been developed in order to enhance both the patient's and caregivers' knowledge of motor and nonmotor PD symptoms. This is intended to allow patients and their caregivers to gain control over their own lives and increase their capacity to recognize, consult opportunely, and solve some important issues at home, thereby facilitating better adaptation to the changes produced by chronic diseases such as PD [12]. The ideal approach to reduce the economic and social burden is a combination between a community approach such as the clinical community and close multidisciplinary collaboration, that is, the integration of medical and nonmedical care and education [13, 14], where the patient and their caregiver play an active role in the creation of a new attention care model for PD.

A clinical community is a group of interdependent individuals united by a shared engagement to specific goals and who work in a synergic manner in order to achieve them. It has a “vertical” integrating core to complement reciprocal “horizontal” relationships between peers [15]. Multidisciplinary teams (MTD) or integrated practice units (IPUs) are “organized around the patient, providing the full cycle of care for a medical condition, including patient education, engagement, follow-up, inpatient, outpatient, and rehabilitative care, and supporting services” [12, 16]. Initiatives such as the Dutch approach called ParkinsonNet are a multifaceted integrative model with clinicians and allied health professionals aiming to improve the multidisciplinary management of PD patients [13]. Thus, MDTs translate collaboration into improved clinical processes [17]. When faced with a suboptimal health system and profound economic limitations, MDT with a community approach becomes almost a necessity; its value in PD patient care has been established in developed countries such as the Netherlands; however, it has not yet been proved in developing countries.

ParkinsonNet was the first reported MTD with an increased quality of care and cost reduction [18]. However, the comprehensive treatment of PD remains high-priced; it requires specialized care [2, 19], and its efficacy has only been tested in developed countries. Currently, in Colombia, as well as many other low- and mid-income countries in Latin-American, there are no government or public health programs for the rehabilitation of PD patients, and given the economic limitations, most patients cannot access a continuous follow-up with neurologists or attend educational programs. Furthermore, according to the direction of epidemiology and demography of the Ministry of Health, in Colombia for 2016-2017, the estimated number of neurologists in the territory was 208 specialists, while the demand for neurology physicians for that same period was 482 [20], making the situation even more critical.

Although there are many questions regarding which of the health interventions are the most cost-effective and adequate for the many existing priorities in the public health systems of low- and mid-income countries, our empirical evidence suggests that integrative health programs implemented in a clinical environment for PD, which practice self-management strategies, may be beneficial for both PD patients and caregivers [21, 22].

This paper aims to document the experience of PD patients, caregivers, and clinical staff who participated in a clinical community approach as an innovative multidisciplinary model in a middle-income setting. This initiative was aimed at patients with PD and their caregivers to empower them in an understanding overview of PD.

### 1.1. Motivation and Background

This initiative began with the creation of Alianza Parkinson in 2013 by an MDT of researchers (clinical and engineers), with the main objective to enhance knowledge about PD through research, education, and empowerment of PD patients and their caregivers, based at Fundación Valle del Lili (FVL), a private, high complexity, university hospital in Cali, Colombia. Considering that QoL relies not only on the opportunity of obtaining an appointment with specialized care, in September 2017, Alianza Parkinson created a program called Saturdays in Motion (SIM), as part of MDT work between the neurology, neuropsychology, and physical therapy departments, intended to improve the QoL of both PD patients and their caregivers. The fundamental pillars of SIM are education, empowerment, and research, supported by PD patients, caregivers, and clinical researchers.

## 2. Materials and Methods

The present research is based on a quantitative descriptive approach. In the following paragraphs, we will describe the process carried out in order to document and report the experience of Saturdays in Motion.

### 2.1. Sampling

The inclusion criteria were having a diagnosis of PD and attending an activity with at least one caregiver (a family member or friend). Thus, we used convenience sampling inviting all PD patients who attended movement disorder consultation at Fundación Valle del Lili, as well as snowball sampling: PD patients invited other PD patients, regardless of their primary consultation centre. SIM has open access to all communities of PD patients, caregivers, relatives, friends, and people from different disciplines related to PD.

### 2.2. Program or Intervention

We used a clinical community model. SIM's primary objective was to create an interdisciplinary approach to PD patients and their caregivers from a medium-income country with low investment in community education and rehabilitation. Secondary objectives include education about the natural course of PD and treatment advances, strengthening the bonds between patient, caregivers, and clinical experts, enhancing the proper home management, building support groups between PD patients and caregivers, and providing support for caregiver burnout. We focus the activities based on constant feedback (questions, suggestions, and personal experiences) from PD patients and caregivers, as well as knowledge from clinical experts. Furthermore, as a result of this integrative model, there is a patient called “patient counsellor,” who is the PD patient's leader and an important communication bridge between the health team and patients.

#### 2.2.1. Session

SIM has been occurring one Saturday per month at Fundación Valle del Lili in Cali, Colombia, since it was established. The activities usually last for approximately four hours per session. The meeting begins with physical and cognitive activation exercises: for example, stretching and exercises aimed at the voice and muscle activation. There are three types of sessions: educational session, cognitive or physical rehabilitation, and leisure activities:

- Educational session: the main seminar is given by an expert on the subject; the daily topic is selected based upon patient and caregiver suggestions from previous sessions, common questions made on clinical consultation with the neurologist, and the knowledge of the clinical variability of PD. Once the seminar has ended, questions are answered, and finally, clinical experts hand out recommendations for patients and caregivers on the home management of the PD symptoms reviewed that day. Also, our clinical leader presents a research report about goals reached and new PD focused challenges for our research team.
- Cognitive or physical rehabilitation: this session is oriented to reinforce the cognitive and physical performance of PD patients. They are guided by a neuropsychologist and occupational therapist and are focused on cognitive exercises such as executive functions, memory, speech, and attention. Physical rehabilitation focuses on balance and swallowing exercises, stretching, and techniques to avoid falling and how to respond if it occurs. Caregivers learn about cognitive activities to share with the patients as well as strategies to avoid falls at home.
- Leisure activities: this session aims to strengthen bonds between all SIM participants, including patients, caregivers, clinical experts, and volunteers. Activities commemorate special days such as St. Valentine's Day, World Parkinson Day, and Christmas. We celebrate with picnics and short run/walk activity, and patients can often show their artistic skills like dancing tango, singing, and declaiming poetry.

### 2.3. SIM Team

We are an interdisciplinary team, composed of physicians (one neurologist, one neurology resident, and a general physician), one physical therapy expert, one neuropsychologist, and volunteers. Most volunteers are undergraduate and master's students from medicine, engineering, and design, who are involved in PD research.

### 2.4. Data Collection

At the end of December 2018 in the final session of the year, we created a survey to evaluate patient and caregiver's perspectives of SIMs usefulness with 16 questions. The objective was to obtain the feedback from patients and caregivers to design SIM activities for 2019. In addition, in July 2019, we applied Parkinson's disease QoL questionnaire (PDQ-39) [23] and Zarit Burden Interview (caregivers self-reported measure) [23] in order to determine the status of the patient's QoL and caregiver's burden, respectively. The scales included our own questions about how SIM influences the QoL of patients and caregivers. This included multiple choice, yes or no, and informative questions. Furthermore, between 2018 and 2019 in order to explore the different perspectives and experiences, we sent two questions by e-mail to clinical experts (see Table 1) from other cities in Colombia and institutional physicians from Fundación Valle del Lili. They were invited to join as speakers. The clinical experts decided to answer by a voice record.

### 2.5. Analysis

We triangulated information from surveys, clinical experts, and previous literature. We also used a descriptive-interpretative approach to describe the design program and the patient, caregiver, and expert's experience. The voice records from clinical experts were heard by three members of SIM teams, and then they were summarized into main topics based on the common points mentioned by the three evaluators. According to data distribution, descriptive statistics were used to summarize the results of the surveys and clinical tests using STATA 13.0.

### 2.6. Ethics Statement

The present research is classified without risk of harm. The Institutional Review Board considered that ethical approval and written consent was not necessary since verbal consent was given prior to interviews and surveys. Furthermore, all information was anonymous (without name and ID).

We guaranteed power balance, reciprocity, and integrity through the active participation of patients and their caregivers. In addition, we were open to listening to their needs, concerns, and expectations and communicated our roles and expectations with honesty.

## 3. Results and Discussion

### 3.1. Results

SIM had an average attendance of 90 subjects including PD patients and caregivers. Most of the attendees do not participate in other multidisciplinary groups. Some PD patients and their caregivers attend SIM monthly. Moreover, there are new patients and caregivers in each session. All surveys were anonymous.

#### 3.1.1. PD Patients

- Sociodemographic data*:* sixty people were invited to answer the survey; 48 people aged between 52 and 92 years answered it completely. Twenty seven were men (56.2%) and 21 were women. About 66.7% of the subjects attended at least six times during the year. Most patients attend with a family member (75%) (see Table 1).
- Activities and QoL*:* the favourite activities were the educational ones (60.4%), followed by stretching exercises (58.3%), group activities (41.6%), and yoga (37.5%). Around 89.5% rated the activities as excellent. 87.9% considered that the activities have improved their QoL. Also, 54.1% wanted SIM activities to be done more often. All patients would recommend SIM to other patients with PD.

**Figure 1:**
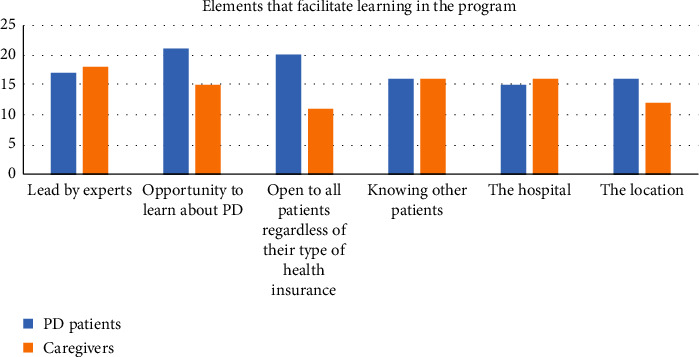
Number of responses about aspects of SIM sessions between PD patients and caregivers.

Most of the patients (95.8%) answered that SIM improved their QoL and is related to an increased awareness of the PD symptoms (87.5%) or the disease itself (79.1%). According to patients, the elements that facilitated learning during SIM sessions were the opportunity to learn about PD outside the routine consultation (87.5%), its accessibility to all patients regardless of their type of medical insurance (83.3%), and the fact that it is led by experts (70.8%) (see Figure 1).

#### 3.1.2. Patient's Quality of Life

- Sociodemographic data: the survey was answered by 36 patients. Only 24 answered it completely, 18 men (70 years, IQR 64–78) and 6 women (66.5 years old, IQR 63–71). The median years since diagnosis were four years (IQR 3–9) (see Table 1).
- PDQ 39: the median of PDQ-39 was 24.6 (±12.7). The most affected QoL dimension was communication (32.2 ± 23.3), followed by body discomfort (34.7 ± 23.3) and emotional well-being and social stigma (21.5 ± 20.2).

#### 3.1.3. Caregiver's SIM Activities and Zarit Scores

**Figure 2:**
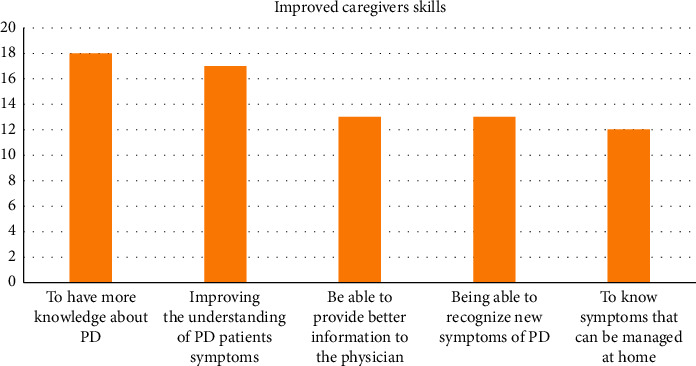
Graph showing the viewpoint of caregivers regarding the most important aspects of the SIM program that could be useful for patient care. Caregivers could select more than one option.

- Sociodemographic data: twenty-one caregivers answered the survey: 20 of them were women, with a median age of 63 years (IQR 55–67). Most frequent caregivers were relatives (90.4%) and spouses (73.6%). In addition to the family members, 42.8% patients had a person who helped them at home.
- Caregivers and QoL: about 85.7% of the caregivers thought that SIM helped them improve their relative's QoL. The factors that facilitated learning in the program were improvement in the understanding of the patient's symptoms (94.4%) and comprehension of the disease itself (85.7%) (see Figure 2).
- Zarit: the median of the Zarit burden interview results was 38 (IQR 31–50). 61.9% of caregivers believed that their QoL was “good.”

#### 3.1.4. Clinical Expert Questions

- Sociodemographic data: the interview was answered by four clinical experts. Neurologist number one is 58 years old and specialized in sleep disorders. Neurologist number two is 38 years old and is an expert in movement disorders. Both neurologists are from Bogotá, Colombia, and the other two were institutional physicians from FVL: one neurosurgeon (44) specialized in functional neurosurgery and one psychiatrist (45) specialized in psychogeriatrics. The psychiatrist was a woman.
- Changing the Traditional Course: regarding the clinical experts perception of PD patients and caregivers, they have “created a community” with people who can understand them, from clinical experts to other PD patients. “Patients and their caregivers are highly motivated,” making this easier for them to continue their treatment. Taking into account the caregiver's role is important because the patient–caregiver relationship can affect the patients and their symptoms: “I have seen an improvement in my clinical consultation, because a tired caregiver generates more symptoms in patients.”

The relevance of this new model of integrated care for PD patients is the course it is taking to change the traditional patient–physician relationship. “SIM has a good structure allowing interaction between patients and clinical experts, which is important because Parkinson's disease affects a lot of aspects including mental and social spheres.” The new model allows a change in the management of PD patients, because despite their limitations due to the disease, SIM “focuses on what patients can still do. Clinical leaders do not limit the program to explain the patient's deficits. Patients and caregivers still feel like there are things to do and things to enjoy.”

At this point, we are grateful for the impact this program is having among clinical experts: “I felt so gratified to see receptive chronic disease patients and receive their feedback” ... “One of my dreams is to have an activity such as SIM in the hospital where I work. Likewise, it is a motivation to reach it at the same level.”

### 3.2. Discussion

This paper aims to document the experience of PD patients, caregivers, and clinical staff who participated in a clinical community approach as an innovative multidisciplinary model in a middle-income setting. This initiative was aimed at patients with PD and their caregivers to empower them in their understanding and overview of PD.

#### 3.2.1. SIM's Community-Based Approach

A community-based approach has many challenges [15], which include the following:

1. Mobilization and encouragement of patients and caregivers to continue in the program: SIM has seen an increase in attendance with a regular group of patients and their caregivers, which attend monthly to SIM's activities. Patients and caregivers are highly motivated as noted by interviews made by clinical experts, and 54.1% of SIM patients would like SIM activities more often. Moreover, reports by Aveling and colleagues [15] state that we have reached patients, caregivers, and clinical staff acceptance without directive or coercive tactics, such as minimum requirements for enrolment or consequences for noncompliance. The patients involved in SIM appreciated the absence of restrictions: “SIM is open to all patients regardless of their type of medical insurance.”
2. Promoting and sustaining a sense of community: SIM creates and promotes bonds between patients, caregivers, and relatives. An iterative topic extracted from clinical interviews was to “be part of a group” and related concepts such as empathy and solidarity. The PD patient's counsellor and his wife have created a personalized support group with other patients. They participate in different activities like periodic visits to the houses of PD patients who cannot come back to SIM, either due to their clinical condition or other difficulties. He expressed that “the group behaves like a family, socializing aspects about the disease and participating in activities such as singing, dancing and declaiming poetry.” Likewise, the PD patient's leader and his wife oversee the Tango dance group. Being a part of a group and sharing experiences could have more benefits than individual-based activities [23]. Accordingly, the clinical community approach might be beneficial for SIM PD patients because they have reported a positive impact on their social activities and emotional well-being improving their self-confidence [24].
3. Moreover, clinical experts talked about the clinical staff–patient relationship in SIM. SIM helps redefine the traditional clinical staff–patient relationship as patients referred to “the opportunity of learning about PD outside the routine clinical consultation.” Neurologist number two and the neurosurgeon also commented that SIM has clinical leaders who created the initiative, organized the budget, and planned activities considering the patient's and caregiver's feedback, their needs, and questions. Consequently, there is a horizontal relationship between all SIM members.
4. Furthermore, groups encourage a peer approach [25] based on empathy. This could benefit treatment and relieve anxiety so far as patients and caregivers recognize that their desires and fears are shared with others, as mentioned by neurologist number one and the psychiatrist, respectively.
5. Sustaining action: SIM has reached two years of monthly sessions thanks to clinical leaders with credibility and legitimacy and the support of one of the most prestigious healthcare institutions in Colombia, Fundación Valle del Lili.

#### 3.2.2. SIM Strengths

SIM aims to enhance the patient's knowledge of motor and nonmotor PD symptoms. PD patients answered that their QoL and knowledge about PD symptoms are directly related to their QoL (87.5%). As shown in the literature [24, 26–28], the enhancement in QoL is frequently reached through activation and education empowerment. The main reason may be that the patients and their caregivers gain control over their own lives and increase their capacity to recognize, consult opportunely, and solve the issues that they often acknowledged as important.

With respect to the other areas of QoL, autonomy and active participation are especially important for PD patients who desire an active involvement in medical decision making and to take control of their life while they are still able [27]. SIM aims to enhance and encourage skills (not only to talk about the deficits) of PD patients, as noted by the psychiatrist. In each session, there is an open space where PD patients can show their abilities to sing, declaim poetry, or dance.

On the other hand, the SIM strategy is not only centred on PD patients; it also takes caregivers into account. Around 66.6% of caregivers reported a mild burden [ZBI 21–40], similar to what has been reported in the literature [29]. This diagnostic finding encouraged us to plan activities dedicated to caregivers. QoL depends greatly on the progression of PD symptoms. In the same way, caregiver's QoL is related to a PD patient's QoL because the overburden that caregivers experience is directly related to the home management quality of PD patients. According to our results, caregivers appreciate “the improvement in the comprehension of patient symptoms,” as SIM is concerned with enhancing the knowledge of caregivers and consequently the home management of PD patients.

Throughout the past two, almost three, years, we have experienced change in the traditional health model, where looking into the information of patients, caregivers, and medical experts, we are building a new integrated patient-centered model. This program has become a reality in a middle-income country, making us aware that this is a continuous work because this approach requires patients' active participation, informed caregivers, and clinical experts open to update (and share) their knowledge, in order to change from hierarchy models to a horizontal approach centered on the empowerment of the patients and caregivers. This initiative agrees with programs such as ParkinsonNet, where the aim roots for the empowerment of patients due to the positive effects on mood symptoms and quality of life, as well as the fact that it makes it less likely to seek medical support and this reduces healthcare costs [30].

#### 3.2.3. Limitations and Future Perspectives

As a descriptive study, the main limitation of this research relates to generalizability. The main objective of our study is to show the experience of SIM in a country with multiple healthcare limitations, like Colombia, in order to provide the first approximation to future investigations about effectiveness in our country. There are limitations regarding sample size; however, it represents a wide range of patients that attend healthcare centres, not only in our institution.

Furthermore, the determination of patient experience and perspective is crucial in order to recognize their needs. In recent research studies, the evaluation of patient-reported outcomes (PROs) has facilitated the selection of variables that are sensitive enough to assess QoL and disability [31]. The development of computerized health applications (apps) can facilitate the use of PROs; this will be considered for the development of new SIM intervention and evaluation strategies.

Currently, in Colombia, as in many other countries in Latin America, there are no specific public health and personalized rehabilitation programs for patients with PD. Due to economic limitations, most patients cannot access continuous follow-up with neurologists or take part in educational programs that could potentially impact their QoL. In contrast to developed countries such as the United States, where 81.9% of homes have access to the Internet [32] (which facilitates information access about the disease to PD patients and their caregivers), only approximately 50% of Colombian households (mainly those in large cities) have Internet access [33]. Our findings indicate that on-site activities with a community approach and a multidisciplinary model may improve the QoL of PD patients and caregivers from urban areas, whilst breaking down social and information barriers. Regardless, future research is needed to confirm this and the remaining questions about these interventions in rural areas with vulnerable communities and different worldviews.

In addition, the progression of PD produces an important disease burden due to functional impairment, not only to patients and caregivers. It has become a challenge for health systems, especially in developing countries, who have limited resources, with many questions lingering around which health interventions are the most cost-effective and adequate for the many existing priorities in public health problems, as demonstrated by ParkinsonNet. Further research is required regarding the cost-effectiveness of SIM. The Dutch model [13] also has education programs for health professionals, which is one of the purposes of our program in the near future.

Despite these limitations, this study represents the first approach to the development of nongovernmental multidisciplinary management teams for Parkinson's disease in Colombia and Latin America.

## 4. Conclusions

Comprehensive and humanized interventions on Parkinson's disease require multidisciplinary assessment with active participation from PD patients and their caregivers. Our preliminary experience shows a multidisciplinary model with a community approach that redefines the relationship between PD patients, their caregivers, and clinical experts. Furthermore, we found a positive perspective about SIM on the potential improvement of PD patients QoL. This will encourage us to do analytic and interventional studies in our country in order to understand the true capacity of this kind of innovative intervention on patient's and caregiver's quality of life as well as the possible advantages of a reduction of health systems' costs.

This study is the first similar work reported in the country, and it is the first time, after more than two years of experience, that we try to formally collect feedback from patients and caregivers. Our results confirm conclusions already documented in previous literature regarding the integrated approach with multidisciplinary teams. This study is the first evidence to show the National Ministry of Health the possible benefits of including this kind of programs as part of the National Health insurance program.

## Figures and Tables

**Table 1:** Surveys and questionnaires given to patients, caregivers, and clinical experts about SIM.

Patients

*Perceptions of SIM*

How many years ago where you diagnosed with Parkinson's disease?
Do you know the program of Alianza Parkinson Cali?
Do you consult our activities on Facebook?
Where did you hear about the activity SIM of the Alianza Parkinson Cali?
With what frequency have you attended the activities of SIM in the last year?
Who goes with you to SIM activities?
Which of the activities on SIM is your favourite?
Which of the following activities do you consider have been the most educative? (mark an X)
How would you rate the quality of the contents of SIM activities?
How satisfied do you feel with the activities of SIM?
Do you consider the activities have improved your quality of life?
Do you consider the activities have increased your knowledge on Parkinson's disease?
Have you practiced the exercises of SIM at home?
Is the space where SIM meetings are held comfortable?
Is the quality of sound good enough to understand everything said in the meetings?
With what frequency would you like the meetings of SIM? Would you recommend the SIM activities to a friend with Parkinson's disease?

*Questions about patients' QoL*

How would you define your quality of life?
Do you consider SIM has contributed in the improvement of your quality of life?
What do you think are the most important elements of the program SIM that contribute the most to improve your quality of life?

Caregivers

*Questions about QoL and burden assessment*

How would you define your quality of life?
Do you consider the time spend with the patient interferes with your quality of life?
Do you consider SIM has contributed in the improvement of the patient's quality of life?
What do you think are the most important elements of the program SIM that contribute the most to improve the patient's quality of life?

Clinical experts

*Experiences on SIM*

What is your point of view on PD patients and caregivers involved in SIM?
How did your participation in SIM change your daily clinical practice?

## Data Availability

The data that support the findings of this study are available upon request to the corresponding author. The data are not publicly available because they contain information that could compromise research participants' privacy/consent.
